# 2251. Evaluating the Financial Implications of Antimicrobial Therapy in Clinical Settings: A Prospective Observational Study

**DOI:** 10.1093/ofid/ofad500.1873

**Published:** 2023-11-27

**Authors:** James Gnanamani, Suresh Kumar Dorairajan, Poojadharshini Magesh, Ratheesh Rajakumar

**Affiliations:** Jaya College Of Paramedical Sciences, Chennai, Tamil Nadu, India; Apollo hospital, Chennai, Tamil Nadu, India; Jaya College of Paramedical Sciences, chennai, Tamil Nadu, India; Jaya College of Paramedical Sciences, chennai, Tamil Nadu, India

## Abstract

**Background:**

Unnecessary antimicrobial use is a significant public health concern that can lead to antimicrobial resistance in hospitalised patients. The WHO defines the rational use of drugs as prescribing the right drug at an adequate dose for an appropriate duration based on the clinical needs of the patient and at the lowest cost possible. Inappropriate use of antimicrobials can lead to drug resistance, medication therapy problems and an increase in drug costs. The aim of this study is to assess the irrational use of antibiotics and to analyse the cost effectiveness of antibiotic prescriptions for patients

**Methods:**

This prospective observational study was conducted in a tertiary care hospital in India for a period of three months. Data on patient demographics and antimicrobial usage were collected including any escalation, de-escalation, discontinuation or other changes to therapy recommended by the hospital's infectious disease specialist (ID) opinion in response to specific patient cases. Follow-up was performed to determine whether the ID opinion recommendations were followed. Financial implications, such as increase in drug costs were documented when the ID opinion was not followed. The necessity of antimicrobial usage and its cost effectiveness were analysed using basic statistical method.

**Results:**

A total of 538 prescriptions for antimicrobial therapy were included in this study. Of these 349 prescriptions (64.8%) were in accordance with ID opinion, while 189 prescriptions (35.2%) were not in accordance with ID opinion. The data was analysed and correlated based on the cost of antimicrobial medication (Table 1)

**Figure:**
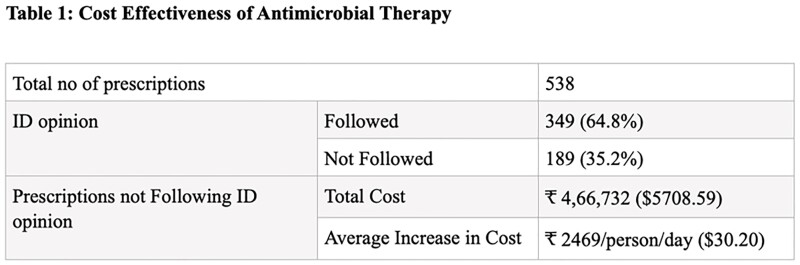

**Conclusion:**

The study found that the average expenditure for a person/day increases by ₹2469 ($30.20) for prescriptions that did not follow ID opinion. This highlights the unnecessary increase in drug expenditure and the risk of antimicrobial resistance. Moreover, these patients had a longer hospital stay on average, leading to additional healthcare costs. Implementing strategies such as antimicrobial stewardship programs and promoting rational use of antibiotics can help reduce unnecessary spending and improve patient outcomes. Further studies are needed to assess the cost-effectiveness of antimicrobial therapy in different clinical settings.

**Disclosures:**

**All Authors**: No reported disclosures

